# Quantifying and Comparing the Match Demands of U18, U23, and 1ST Team English Professional Soccer Players

**DOI:** 10.3389/fphys.2021.706451

**Published:** 2021-07-02

**Authors:** James Reynolds, Mark Connor, Mikael Jamil, Marco Beato

**Affiliations:** School of Health and Sports Science, University of Suffolk, Ipswich, United Kingdom

**Keywords:** football, team sports, GPS, speed, performance

## Abstract

The aim of this study was to quantify and compare the match load demands of U18, U23, and 1ST team players during the official season. A total of 65 matches and 495 (U18 = 146, U23 = 146, and 1ST team = 203) individual player game observations were included in this analysis. A 10-Hz global navigation satellite systems (GNSS) and 100-Hz triaxial accelerometer (STATSports, Apex, Northern Ireland) were used to monitor the following metrics during official matches: total distance, high-speed running distance (HSR), sprint distance, high metabolic distance, explosive distance, high-intensity bursts distance, speed intensity, and dynamic stress load (DSL) were analyzed. A multivariate analysis of variance test reported significant (*p* < 0.001) differences among the groups. HSR during matches was lower (*d* = *small*) for U18 players than the U23 and 1ST team players. Sprint distance and high-intensity bursts distance were lower (*small*) in U18 compared with the U23 and 1ST team. DSL was greater in 1ST compared with U18 (*small*) and U23 (*small*). This study reported that the differences between groups were greater for HSR, sprint distance, high-intensity bursts distance, and DSL, while total distance, high metabolic load distance, explosive distance, and speed intensity did not differ between the groups. These findings could be used to design training programs in the academy players (i.e., U18) to achieve the required long-term physical adaptations that are needed to progress into the U23 and 1ST teams.

## Introduction

Soccer players need to be adequately trained to cope with the high physical demands, such as sprints, high-speed running distance (HSR), accelerations, and decelerations, they experience during an official match (Mohr et al., 2005; Gualtieri et al., 2020). In recent years, the analysis of external training load has become one of the most important tasks for sport science departments (Akubat et al., 2014). This type of objective data can facilitate the training decision process of sport science staff and coaches during the soccer season (Gualtieri et al., 2020). Training load analysis is commonly analyzed using global navigation satellite systems (GNSS) (Cummins et al., 2013; Beato et al., 2018b). The adequate application of training load monitoring procedures and consequent training planning can have a critical impact on the players’ readiness and long-term fitness status (Vanrenterghem et al., 2017; Chmura et al., 2019). These factors are important in professional soccer where teams have hectic schedules that can limit the time available for physical training and recovery (e.g., travel commitments, need for tactical skills, and technical training) (Beato et al., 2019a; Gualtieri et al., 2020). Previous research provided evidence that the match has an important impact on physical adaptations and is the most demanding session of the week (Morgans et al., 2018). Therefore, coaches and sports scientists need to adequately monitor training load during the match to ensure the right balance of training and recovery are prescribed to the players during a microcycle and throughout the entire season (Vanrenterghem et al., 2017). For these reasons, comprehensive research and analysis are required to determine the match load demands and relevant outputs of differing age groups (e.g., U18, U23, and 1ST team).

In the last decade, an increase in match physical and technical performance parameters in professional soccer has been reported (Bush et al., 2015; Bradley et al., 2016). This information allows sports scientists and coaches to design training drills to appropriately expose players to match like running conditions (e.g., intensity) (Konefał et al., 2019; Gualtieri et al., 2020). This is particularly important because academy players (U18) need to be physically fit to move up into the U23 squad and into the 1ST team (Barnes et al., 2014; Murtagh et al., 2018). It is generally supposed that a difference in the match demands and physical output between these groups (U18, U23, and 1ST team) exists; however, direct comparisons between squads and age groups within the same professional club is currently missing from the research literature. In particular, there is limited information concerning U18 and U23 match loads, while 1ST team matches have been frequently investigated (Rampinini et al., 2009; Bush et al., 2015). The explanation for such a discrepancy of information between U18, U23, and 1ST team players may be due to the shortage of monitoring technology in academy squads, explained in part by the high cost of this technology, which limits the ability of some clubs to conduct match demands-based research. The analysis of match load between these squads may help sports science departments to better understand the differences that exist between these groups and, therefore, to design the training programs in the academy to achieve the required long-term physical adaptations that are needed for physical development and for player progression from U18 to the 1ST team. Therefore, the aim of this study was to quantify and compare the match load demands of each of academy U18, U23, and 1ST team players during the official season.

## Methods

### Participants

A total of 67 male professional soccer players of the same club were enrolled in this study. The inclusion criteria were the absence of illness and injuries and regular participation in soccer competitions. Goalkeepers were excluded by this study and only outfield players match data were evaluated. The sample size power was evaluated using G^∗^power (Düsseldorf, Germany), and results indicated that a total sample of 48 participants would be required to detect a *moderate* effect (*f* = 0.35) with 80% power and an alpha of 5%. External training load data were recorded as part of the normal monitoring routine of the club and was analyzed *a posteriori*. The Ethics Committee of the University of Suffolk (Ipswich, United Kingdom) approved this study (RDU21/008). Informed consent to take part in this research was signed by the players. All procedures were conducted according to the Declaration of Helsinki for human studies.

### Experimental Design

Players were divided into U18 team (19 players), U23 team (17 players), and 1ST team (20 players). Only players who played for the full duration of the match were included in this analysis. A total of 65 matches and 495 (U18 = 146, U23 = 146, and 1ST team = 203) individual player game observations were included in this analysis.

### Global Navigation Satellite Systems and Data Recording Procedure

External match data were recorded during official competitions by the 10-Hz GNSS system and 100-Hz triaxial accelerometer (STATSports, Apex, Northern Ireland). GNSS technology is capable of acquiring and tracking multiple satellite systems (e.g., global positioning systems, GLONASS) to provide the most accurate positional information (Beato et al., 2018a). These GNSS units have been previous validated for both linear and sport-specific distance – bias 1–2.5% (Beato et al., 2018a). The inter-units’ reliability was *excellent* (intra-class correlation coefficient = 0.99), with a typical error of measurement of 1.85% for sprint ranging from 5 to 30 m (Beato and De Keijzer, 2019). The units were turned on about 15 min before the beginning of the data recording. The Apex GNSS model reports information about the quality of the signals, which ranged between 16 and 21, which is in line with previous literature (Beato and De Keijzer, 2019). All data recorded by the GNSS units were downloaded and processed using the STATSports Software (Apex version 3.0.02011) before being exported to CSV for further analysis.

### External Load Variables

Total distance covered measured in meters and HSR over 5.5 ms^–1^ (19.8 km h^–1^) and sprinting distance over 7.0 ms^–1^ (25.2 km h^–1^) measured in meters were analyzed (Beato et al., 2020). High metabolic load distance (value of 25.5 W kg^–1^) measured in meters were analyzed by di Prampero’s model (di Prampero and Osgnach, 2018). Explosive distance is defined as the distance (m) covered by a player when their metabolic power is above a threshold of 25.5 W kg^–1^, but their velocity is below a HSR threshold of 5.5 m s^–1^ (19.8 km h^–1^). High-intensity bursts distance is measured in meters, which is defined as any three high-intensity activities (acceleration ≥ 4.0 ms^–2^, deceleration ≤ −4.0 m s^–2^, or impacts ≥ 11G) completed in succession separated by 20 s or less. Speed intensity is measured in arbitrary units (AU), which is a measure of total exertion calculated as the sum of a convexly weighted measure of instantaneous speed. Dynamic stress load (DSL) is an accelerometer-derived metric which aggregates the rates of accelerations on its three orthogonal axes (*X*, *Y*, and *Z* planes) to form a composite magnitude vector (expressed as G force) which this inputted to a curved weighted function to get a value in AU (Beato et al., 2019b).

### Statistical Analyses

Descriptive statistics are reported as mean ± SD. A multivariate analysis of variance test was used to assess if significant differences exist between groups across several dependent variables. A Shapiro–Wilk test was used to check the assumption that the data conform to a multivariate normal distribution, where significant a multivariate power transformation has been applied. A series of univariate one-way ANOVA tests were conducted for each dependent variable to evaluate between-group differences. When significant differences were found, *post hoc* analysis was performed using Bonferroni corrections, estimates of 95% CI were calculated using a bootstrapping technique (1,000 random bootstrap samples) and effect sizes were reported using the Omega squared method to correct for variance bias. Effect sizes were interpreted using Cohen’s *d* principle as follows: *trivial* < 0.2, *small* 0.2–0.6, *moderate* 0.6–1.2, *large* 1.2–2.0, and *very large* > 2.0 (Hopkins et al., 2009). Unless otherwise stated, significance was set at *p* < 0.05 for all tests. Statistical analyses were performed in JASP (JASP version 0.14.1, Amsterdam, Netherlands).

## Results

Summary of the U18, U23, and 1ST team match loads is reported in Table 1.

**TABLE 1:** Summary of the U18, U23, and 1ST teams’ match loads.

Variable	U18 mean ± SD	U23 mean ± SD	1ST team mean ± SD
Minutes played (min)	95 ± 3	94 ± 3	96 ± 2
Total distance (m)	10,259 ± 883	10,052 ± 715	10,141 ± 835
High-speed running distance (m)	626 ± 228	704 ± 217	673 ± 249
Sprint distance (m)	110 ± 82	142 ± 82	144 ± 89
High metabolic load distance (m)	2,034 ± 386	2,062 ± 330	1,990 ± 410
Explosive distance (m)	1,408 ± 300	1,358 ± 226	1,317 ± 260
High-intensity bursts distance (m)	406 ± 217	488 ± 259	585 ± 320
Speed intensity (AU)	505 ± 53	496 ± 46	499 ± 55
Dynamic stress load (AU)	346 ± 164	323 ± 133	516 ± 267

The results of the multivariate analysis test for the group analysis were *F* = 14.020, *Trace*_Pillai_ = 0.467, and *p* < 0.01.

The results of the individual ANOVA analysis tests are detailed in Table 2.

**TABLE 2:** U18, U23, and 1ST team match day training load univariate comparisons.

Variable	*F*	*P* value	Group		*Post hoc* (Bonferroni)	95% bca CI		Effects size (Cohen’s d)	Qualitative assessment
Total distance (m)	0.461	0.631	−		−	−		−	−
High-speed running distance (m)	3.498	0.040*	1ST	U18	1.000	−4.176	3.886	0.003	*Trivial*
				U23	0.074+	−8.011	0.293	0.263	*Small*
			U18	U23	0.096+			0.272	*Small*
						−7.930	0.314		
Sprint distance (m)	4.501	0.011*	1ST	U18	0.047*	0.154	1.937	0.277	*Small*
				U23	1.000				
						−1.150	0.821	0.059	*Trivial*
			U18	U23	0.015*	−2.261	−0.351	0.347	*Small*
High metabolic load distance (m)	2.542	0.080	−		−	−		−	−
Explosive distance (m)	2.801	0.126	−		−	−		−	−
High-intensity bursts distance (m)	5.728	0.004**	1ST	U18	0.003**	1.205	4.503	0.396	*Small*
				U23	0.741	−0.700	2.552	0.132	*Trivial*
			U18	U23	0.089+	−3.348	0.268	0.275	*Small*
Speed intensity (AU)	0.617	0.540	–		−	−		−	−
Dynamic stress load (AU)	14.693	<0.001***	1ST	U18	<0.001***	0.024	0.056	0.587	*Small*
				U23					
					<0.001***	0.023	0.057	0.505	*Small*
			U18	U23	1.000	−0.016	0.017	0.035	*Trivial*

95% CIs are reported as Box-Cox transformed values for the difference between pairwise group means. 1ST = senior team; AU = arbitrary units. Significant level: +*p* < 0.1; *p < 0.05; **p < 0.01; and ***p < 0.001.

## Discussion

The aim of this study was to quantify and compare the match load demands of U18, U23, and 1ST team players during the official season. 1ST and U23 groups reported higher match demands compared with U18 players in sprinting distance, high-intensity bursts distance, and DSL. However, total distance, high metabolic load distance, explosive distance, and speed intensity did not differ among the teams. U23 players reported lower DSL and equivalent sprinting distance, respectively, compared with the 1ST, while HSR was greater (*d* = *small*) compared with both the U18 and 1ST teams. Soccer practitioners could compare the findings reported in this study with the match demands of their academy and 1ST players; based on the results of this study, they may wish to focus their attention on monitoring sprinting distance, HSR distance, high-intensity bursts distance, and DSL, which have been shown to discriminate between the academy and 1ST team players; however, because this analysis was performed enrolling only the players of one club, wide generalization to other teams cannot be performed. The 1ST team and U23 team reported very similar match load demands, apart from DSL. The differences reported in match demands in this study should be also considered when developing the physical qualities needed to progress from U18 to the U23 and 1ST teams.

Sports scientists need to monitor the training and match loads of their players to balance and plan appropriate physical stimuli during training sessions (Vanrenterghem et al., 2017; Connor et al., 2021). Several researchers reported that the match represents the most important physical stimulus of the week and plays a key role in achieving long-term physical development (Anderson et al., 2016; Morgans et al., 2018; Gualtieri et al., 2020). This study reported normative match data of age groups of professional players (Table 1) and the differences that exist between these groups (Table 2), which can be very important for practitioners and sports science departments to have a better overview of physical demands from academy to 1ST team. Our analysis showed that U18 players generally perform less physical activity than U23 players and 1ST team players in some but not all the metrics analyzed (Table 2). HSR during matches was reported to be lower (*small*, *p* = 0.096) for U18 players than U23 players. Sprint distance reported *small* (*d* = 0.347 and 0.277) differences between U18 and U23 and 1ST teams, respectively. U23 players reported very similar external load parameters compared with the 1ST team – except for greater (*small*, *p* = 0.074) HSR distance. Previous research has clearly shown that sports scientists and coaches should evaluate the match demands of their players to replicate the same intensities during training (Dello Iacono et al., 2019). Based on this research, we have shown the importance of quantifying match demands across the varying playing levels to objectively quantify the existing differences. This approach can offer useful insights to coaches and practitioners, who should replicate the analysis reported in this study and use the resulting data to design the most suitable training sessions and adopt the most ecological drills to obtain the long-term physical adaptations needed to progress from an academy squad (i.e., U18) to an U23 or 1ST team (Beato et al., 2019a; Dello Iacono et al., 2019). In this study, we have found that high-intensity metrics such as HSR (significant group differences reported in the ANOVA but not following the *post hoc* analysis) and sprinting can discriminate between age groups as well as high-intensity bursts distance; therefore, sport scientists may include these metrics when monitoring and planning sport-specific drills, which can be beneficial to enhance the performance capacities required during a match (Dello Iacono et al., 2022). The importance and the rationale for the monitoring and implementation of HSR and sprinting have been recently discussed in detail (for further in-depth consideration, please see Beato et al., 2020). Furthermore, DSL, which is an accelerometer-derived metric that aggregates the rates of accelerations on its three orthogonal axes (Beato et al., 2019b), reported a *small* difference between 1ST team players (515 AU) compared with U18 (346 AU) and U23 (323 AU); instead, total distance, high metabolic load distance, explosive distance, and speed intensity performed during matches were not different among groups. The similarity in total distance between teams could be explained in part by the nature of this metric, which indicates the volume of running covered during a match, which simply may not discriminate between teams and different running intensities with the same sensitivity as other metrics do (e.g., sprinting distance). The total distances reported in this study are in line with previous research analyzing soccer players (i.e., 10,551 ± 974) (Morgans et al., 2018). Authors may explain the similarity in high metabolic load distance, explosive distance, and speed intensity between teams by considering the between-match variability of physical performance (match contextual factors) (Carling et al., 2016; Lorenzo-Martínez et al., 2020). The observed differences were not significant between the teams possibly because of the variability of these metrics between matches, which could be due to factors not considered in this study such as situational and environmental factors (Trewin et al., 2017); future investigation may evaluate the external load difference that exists between squads enrolling a larger sample of participants that may increase the statistical power of the analysis to verify our results. Based on our findings, we suggest to soccer practitioners to consider the monitoring and subsequently designing of training sessions based on HSR and sprinting data – which can discriminate match running performance among teams; however, we recommend replicating the analysis performed in this study to verify the match demands of their academy and 1ST players. Moreover, practitioners may consider the monitoring of high-intensity bursts distance and DSL. Previous research has shown that DSL can quantify players’ mechanical load (Beato et al., 2019b); however, further research is needed to verify the sensitivity of this metric to differentiate among age groups and teams’ levels.

This study is not without limitations. First, a single club was analyzed in this study and therefore the players and the three age groups studied represent a unique sample. This unique characteristic could limit the application of our findings to other clubs, but the enrollment of teams within the same club has limited the possible confounding factors associated with different types of facilities, player quality, and technologies used to monitor the match load, which could have affected the ecological validity of this research. The second limitation is related to the GNSS technology, which presents some inaccuracy and therefore practitioners should consider that external load data may present an error (generally ranging between 1 and 2.5%). This study limited the effects of this, and in particular errors related to inter-model variability, as all players used the same GNSS units that received previous validation (Beato et al., 2016, 2018a; Beato and De Keijzer, 2019). Lastly, this study did not analyze the difference in external load parameters between playing positions, which has been reported before to be a discriminant factor (Rampinini et al., 2007). Future studies may replicate the analysis reported in our study at a positional level alongside other contextual factors.

## Conclusion

This study quantified and compared the match load demands of U18, U23, and 1ST teams during the official season reporting that U18 players performed significantly lower match load than U23 and 1ST team, but in not all the metrics. Instead, the 1ST and U23 team players generally performed similar match load during competitions. This study reported that the differences between groups existed for sprint distance, high-intensity bursts distance, HSR, and DSL, while total distance, high metabolic load distance, explosive distance, and speed intensity did not differ between the groups. These findings could be used to design training programs in the academy players (i.e., U18) to achieve the required long-term physical adaptations that are needed to progress into U23 and 1ST teams.

## Data Availability Statement

The raw data supporting the conclusions of this article will be made available by the authors, without undue reservation.

## Ethics Statement

The studies involving human participants were reviewed and approved by University of Suffolk, Ipswich, United Kingdom. Written informed consent to participate in this study was provided by the participants’ legal guardian/next of kin.

## Author Contributions

JR collected the data used in this research. MC and MJ performed the statistical analysis and reported the results. MB coordinated the project. All authors were involved in the writing of the manuscript.

## Conflict of Interest

The authors declare that the research was conducted in the absence of any commercial or financial relationships that could be construed as a potential conflict of interest.
